# Usefulness of Home Overnight Pulse Oximetry in Patients with Suspected Sleep-Disordered Breathing

**DOI:** 10.1155/2020/1891285

**Published:** 2020-11-12

**Authors:** Cristina Esteban-Amarilla, Silvia Martin-Bote, Antonio Jurado-Garcia, Ana Palomares-Muriana, Nuria Feu-Collado, Bernabe Jurado-Gamez

**Affiliations:** ^1^Department of Respiratory Medicine, Pitie Salpetriere University Hospital, Paris, France; ^2^Department of Respiratory Medicine, Infanta Leonor University Hospital, Madrid, Spain; ^3^Physiotherapy Unit, San Juan de Dios Hospital, Cordoba, Spain; ^4^Department of Respiratory Medicine, Hospital de Alta Resolución, Puente Genil, Cordoba, Spain; ^5^Department of Respiratory Medicine, Reina Sofía University Hospital, Córdoba, Spain; ^6^Maimónides Biomedical Research Institute of Cordoba (IMIBIC), Córdoba, Spain; ^7^School of Medicine, University of Córdoba, Córdoba, Spain

## Abstract

**Methods:**

Prospective study conducted in a university hospital. Subjects with a clinical suspicion of SAHS were included. All of them underwent home polygraphy and oximetry on the same night. A correlation was made between the apnea-hypopnea index (AHI) and the oximetry variables. The variable with the highest diagnostic value was calculated using the area under the curve (AUC), and the best cut-off point for discriminating between patients with SAHS and severe SAHS was identified.

**Results:**

One hundred and four subjects were included; 73 were men (70%); mean age was 52 ± 10.1 years; body mass index was 30 ± 4.1, and AHI = 29 ± 23.2/h. A correlation was observed between the AHI and oximetry variables, particularly ODI3 (*r* = 0.850; *P* < 0.001) and ODI4 (*r* = 0.912; *P* < 0.001). For an AHI ≥ 10/h, the ODI3 had an AUC = 0.941 (95% confidence interval (CI) = 0.899–0.982) and the ODI4, an AUC = 0.984 (95% CI = 0.964–1), with the ODI4 having the best cut-off point (5.4/h). Similarly, for an AHI ≥ 30/h, the ODI4 had an AUC = 0.922 (95% CI = 0.859–0.986), with the best cut-off point being 10.5/h.

**Conclusion:**

Nocturnal oximetry is useful for diagnosing and evaluating the severity of SAHS. The ODI4 variable was most closely correlated with AHI for both diagnosis.

## 1. Introduction

Sleep apnea-hypopnea syndrome (SAHS) is a major public health problem due to its high prevalence, which can reach up to 25% among middle-aged adults [1]. It is also associated with increased vascular morbidity and mortality [2, 3], and fragmented sleep results in nonrestorative sleep and excessive daytime sleepiness. It is important to make an early diagnosis, as continuous positive airway pressure (CPAP) treatment is effective for controlling SAHS and vascular risk factors [4, 5]. Full-night polysomnography is currently considered the gold standard for sleep studies [6], although it is limited by a lack of availability in all hospitals, long waiting lists, and high cost. Cardiorespiratory polygraphy, a technique validated for the diagnosis of SAHS, is therefore increasingly used [7–9]. Nevertheless, SAHS is still highly underdiagnosed, and this has sparked interest in other more simplified alternative techniques. Nocturnal oximetry requires no previous training, correctly detects peripheral oxygen saturation (SpO_2_), and shows a typical pattern of intermittent hypoxemia in patients with SAHS. For these reasons, it has been used in both sleep laboratories [10] and home [11–14]. Moreover, it could be a tool available to primary care medicine. Results obtained with this technique, however, are highly variable [13], and it is not recommended as a general practice in clinical practice guidelines [15]. Some authors have suggested that nocturnal oximetry could be a diagnostic alternative in subjects with symptoms of SAHS and could also aid in therapeutic decision-making [10, 11]. However, these aspects have not been well studied in clinical practice conditions. In this study, the main aim was to assess the diagnostic yield of home nocturnal oximetry versus polygraphy in patients with symptoms suggestive of SAHS.

## 2. Materials and Methods

### 2.1. Design

This is an investigator-blinded prospective study comparing diagnostic tests and evaluation.

The protocol for this study was approved by the Cordoba Research Ethics Committee (ref: 2965).

### 2.2. Study Population

Individuals with clinical suspicion of SAHS were assessed in the sleep unit of a university hospital. Individuals who consecutively referred to the sleep unit for polygraphy and who met the following criteria were included: (i) aged between 18 and 75 years; (ii) willing and able to perform the diagnostic tests in the home; (iii) signed informed consent. The exclusion criteria were (i) thoracogenic (severe kyphoscoliosis) or neuromuscular disease, or chronic respiratory failure resulting from any cause (breathing room air, an SpO_2_ < or = 92%), (ii) drug user, including alcoholism, and (iii) specific sleep disorder other than respiratory disorders, especially insomnia.

### 2.3. Methods

All patients who met the inclusion criteria underwent home nocturnal oximetry and polygraphy on the same night, following clinical practice guidelines [6]. The following variables were recorded of patient history: sex, body mass index (BMI), alcohol and medication use, smoking habits, general, and sleep-related symptoms (snoring, gasping, witnessed apnea, nocturia, nonrestorative sleep, morning headaches, and excessive daytime sleepiness), comorbidities (diabetes, dyslipemia, vascular disease, hypertension, and cardiac arrhythmia), and respiratory diseases other than SAHS (bronchial asthma and chronic obstructive pulmonary disease (COPD).

Polygraphy was carried out in the patient's home (Somnea®, Compumedics Ltd., Abbottsford, Australia) following the established recommendations [6, 16]. The methodology has been previously described and validated in our sleep unit [11]. Briefly, all the studies included recording of the oronasal flow and pressure, respiratory movements, and SpO_2_. Apnea was defined as a significant decrease (>90%) in oronasal flow of >10 s and hypopnea as an evident decrease in airflow >30%, but <90%, and associated with either oxygen desaturation of ≥3%. The variables analyzed were the apnea-hypopnea index (AHI), defined as the number of apneas plus hypopneas per hour of recording, baseline peripheral oxygen saturation (SpO_2_), mean SpO_2_, minimum SpO_2_, oxygen desaturation index (OID3 or ODI4), defined as the number of times SpO_2_ fell by ≥3% or 4%, respectively, and the T90 or percentage time recorded with SpO_2_ < 90%. The test was inconclusive if there was a >25% loss in basic signals (respiratory flow and SpO_2_). Clinically relevant SAHS was diagnosed if the test showed an AHI ≥ 10/h and severe SAHS if the AHI was ≥30/h [6, 15].

Nocturnal oximetry was performed with a pulse oximeter (Konica-Minolta Pulsox-300i; Software: Data Analysis DS-5). According to the manufacturer's recommendations, the sampling frequency of the Pulsox-300i is set at 1 Hz with an averaging duration of 3 sec and a resolution of 0.1% SpO_2_. Absence of variability in the SpO_2_ measurement in the index finger of each hand was verified in the sleep unit, and the patient was taught how to place the pulse oximeter. Oximetry data were automatically scored and then visually checked by the physician, and obvious artifacts in the oximetry signal were excluded.

Manual interpretation of the polygraph and oximetry was always performed by the same physicians, and the patient's information was anonymized by means of an alphanumeric code, thus preventing the investigator from influencing the results.

### 2.4. Statistical Analysis

Categorical variables are expressed as absolute numbers and percentages, and statistical significance was tested using the Chi^2^ test or Fisher's exact test. To compare the severity groups, the Kruskal–Wallis test was used. Continuous variables are described using the mean ± standard deviation (SD) for data with normal distribution, and statistical comparison for continuous data with normal distribution was performed using the Student's *t*-test. After verifying that the data had a normal distribution, the Pearson correlation between the AHI obtained from the polygraph and those observed in the oximetry was analyzed, after which a receiver operator characteristic (ROC) curve was constructed and the area under the curve (AUC) was calculated for the overnight SpO_2_ variables to determine which were more accurate in diagnosing AHI ≥ 10/h and AHI ≥ 30/h. All statistical tests were two-tailed, accepting a *P* value <0.05 as statistically significant. Data were analyzed using the Statistical Package for Social Sciences (SPSS 15.1) for Windows (SPSS, Chicago, IL, US).

## 3. Results and Discussion

### 3.1. Results

A total of 119 subjects were included, 15 of whom were excluded (13%), 5 due to inconclusive polygraphy (4%) and 10 due to inconclusive oximetry (8%). The final sample was therefore composed of 104 subjects: 74 (71%) were classified as SAHS, while 39 (37%) presented with severe SAHS (AHI ≥ 30/h). Following the Global Initiative for Chronic Obstructive Lung Disease 2017, COPD patients showed mild (*n* = 16), moderate (*n* = 5), severe (*n* = 1), and very severe (*n* = 0) functional grade, with the latter patient having FEV_1_ = 44% and a baseline SpO_2_ of 95%. The baseline characteristics of the study subjects and symptoms are shown in Tables 1 and 2.

**Figure 1:**
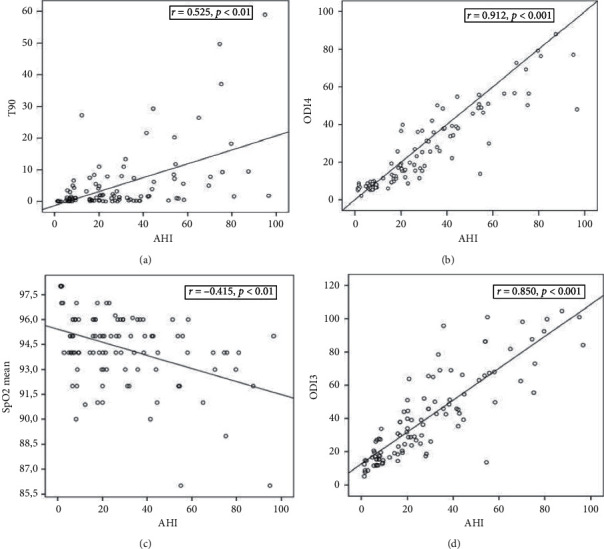
Correlations of apnea-hypopnea index with oxygen desaturation index and percentage of recording with SpO_2_ < 90% measured by nocturnal oximetry. The correlation in ODI3 (good correlation, *r* > 0.8) and ODI4 (excellent correlation, *r* > 0.9) is shown. AHI: apnea-hypopnea index; ODIs: mean number of oxygen desaturations ≥3% and 4% (ODI3 and ODI4) per hour of recording; T90: time spent with SpO_2_ < 90%.

The Pearson statistic was applied in order to determine the correlation between the AHI values and the pulse oximetry variables. The results are shown in Figure 1 and, as can be seen, the nocturnal SpO_2_ variables showed a significant correlation with AHI. Using the 3 variables that showed the best correlation, a ROC curve was constructed using the AHI observed in the polygraph to evaluate the diagnosis of SAHS (AHI ≥ 10/h) and severe (AHI ≥ 30/h). The results are shown in Table 3.

**Figure 2:**
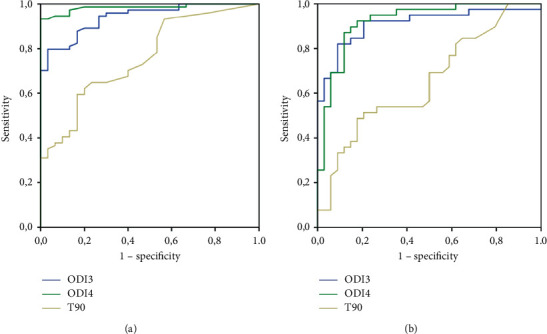
Receiver operating characteristic curves for oxygen desaturation index and percentage of recording with SpO_2_ < 90% with thresholds. (a) AHI ≥ 10/h and (b) AHI ≥ 30/h, respectively. The discrimination degree between AHI ≥ 10/h and ODI3 (very good, AUC = 0.941) and ODI4 (excellent, AUC = 0.984) (a) and between AHI ≥ 30/h and ODI3 (very good, AUC = 0.911) and ODI4 (very good, AUC = 0.922) is shown. AHI: apnea-hypopnea index; ODIs: mean number of oxygen desaturations ≥3% and 4% (ODI3 and ODI4) per hour of recording; AUC: area under the curve.

As can be seen in Figure 2(a), the ODI4 had the best diagnostic accuracy for a sensitivity = 1 and a specificity > 85%; the cut-off point was 5.4/h. For an AHI ≥ 30/h, in Figure 2(b), the ODI4 also showed good diagnostic validity; the best cut-off point was 10.5/h for a sensitivity = 1 and a specificity > 85%.

## 4. Discussion

We evaluated the role of home nocturnal oximetry versus polygraphy in patients referred for suspected SAHS. The main study findings were that the ODI4 and ODI3 measured by nocturnal oximetry showed a correlation with AHI. Therefore, results suggested a diagnostic approximation defined by the gold standard for SAHS [6, 16] and also provide relevant information for therapeutic management of severe SAHS and CPAP indication [6]. These results are clinically applicable, given the availability and ease of use of home overnight pulse oximetry. In clinical practice, polygraphy is a diagnostic tool used extensively in the patient's home [7–9] if there is clinical suspicion of sleep-disordered breathing and is ideal as a reference for comparison with home nocturnal oximetry. The variability in the nocturnal oximetry methodologies used in different studies must be considered when comparing our results with previous findings [10–14, 17]. In some studies, nocturnal oximetry was not done on the same night as the sleep test (polysomnography or polygraphy) [12, 13, 17], while in others, the tests were carried out in a sleep laboratory [11, 18, 19]. These differences, therefore, make it impossible to generalize the conclusions of these authors to the out-of-hospital environment. In order to avoid variability in the results between different nights, in our study, both polygraphy and nocturnal oximetry were performed on the same night. It is also relevant to highlight the importance of the significant desaturation criterion [19]; Ho et al. [20] showed that the percentage decrease in SpO_2_ used to define hypopnea significantly influences the diagnosis and severity classification of SAHS. This could explain the variations in sensitivity and specificity of nocturnal oximetry compared to polygraphy. In our study, we followed current recommendations for the interpretation of polygraphy results [6, 16] and defined hypopnea as a ≥3% drop in SpO_2_ [6, 21, 22]. Taking into account the AHI observed in the polygraph, the ODI4 gave the most accurate diagnosis and severity classification of SAHS in the nocturnal analysis of SpO_2_. However, the ODI3 also shows a high diagnostic performance. Both parameters have more than a 90% probability that the diagnosis made in patients with SAHS is correct. Therefore, it would allow prioritizing the diagnosis in patients with SAHS and in those with severe SAHS, starting treatment with CPAP [6]. This is a key finding and could help define overall strategies for the management SAHS. The specificity increases in patients with moderate-to-severe disease [11–13], although it also correlates well in subjects with mild-to-moderate SAHS [23]. It has been observed that an OID4 > 15 has a positive predictive value of 100%, which could avoid having to perform sleep tests [10–13]. In these studies, the criterion for classifying hypopnea was a drop in SpO_2_ > 4%. However, our work shows that the ODI3 is also equally useful to correctly classify patients with SAHS and with severe SAHS. In addition, the ODI and the T90 report on the hypoxemic burden in the patient with SAHS and correctly determine intermittent hypoxemia [10–13, 17]. Desaturation severity predicts all-cause and cardiovascular mortality [24, 25]. Therefore, the additional information on SpO_2_ is clinically important and may modify the therapeutic strategy in patients with severe OSA and vascular risk factors or advanced age. These results are consistent with our findings and support the use of overnight pulse oximetry when SAHS is suspected. In a recent study, the authors concluded that, for a disordered breathing rate of 15/h, an OID4 ≥ 7/h obtained in pulse oximetry had a positive predictive value of 97% [13]. As previously mentioned, in our study, the ODI4 provided the best yield for diagnosing the severity of SAHS (AHI ≥ 30/h), however, with little advantage over the ODI3. When included in a clinical protocol, this finding would be an indication for CPAP treatment to be initiated, followed by split-night polysomnography. It should be remembered that diagnosis and treatment is very important in vascular disease [2–5], occupational health [26], and elderly men [25]. In these circumstances, nocturnal oximetry may be useful for prioritizing polygraphy or polysomnography studies or for identifying patients with SAHS and indicating the best therapeutic strategy on an individual basis. We have shown that home nocturnal oximetry can be useful in clinical practice for the diagnosis and treatment of SAHS, always taking into account the patient's medical history and signs and symptoms that could indicate a higher risk of SAHS [27]. Furthermore, the low cost, ease of use, and availability of pulse oximetry make it a good alternative in the primary care setting [28, 29]. The study had some limitations. It was conducted in a tertiary reference hospital, and subjects were previously assessed in a specific sleep clinic, so the results should be limited to this clinical context.

## 5. Conclusions

In conclusion, in patients with suspected SAHS, home nocturnal oximetry shows acceptable correlation with AHI determined by polygraphy. The variable with best capacity for discriminating between subjects with or without SAHS and establishing severity was the ODI4. These results therefore support the home nocturnal oximetry, especially in primary care medicine, which could be a screening tool for the diagnosis of SAHS, helping therapeutic management.

## Figures and Tables

**Table 1:** Demographic and clinical information for the 104 subjects included in the study.

	Values
Age (years)	52 ± 10.1
BMI (kg/m^2^)	30 ± 4.1
Male (number, %)	73 (70%)
Epworth sleepiness scale	9 ± 4.1
Diabetes (number, %)	23 (22%)
COPD (number, %)	22 (21%)
Dyslipidemia (number, %)	34 (33)
Vascular disease (number, %)	22 (21%)
Hypertension (number, %)	49 (47%)
Cardiac arrhythmia (number, %)	14 (13%)
AHI (events/h)	28 ± 23.2
Baseline SpO_2_ (%)	95 ± 1.6
Mean SpO_2_ (%)	94 ± 2.2
ODI3 (events/h)	40 ± 26
ODI4, (events/h)	25 ± 20.4
T90, (%)	5 ± 9.7

Results are presented as mean ± standard deviation for continuous variables and number (%) for categorical variables. BMI: body mass index; COPD: chronic obstructive pulmonary disease; AHI: apnea-hypopnea index; SpO_2_: oxygen saturation measured by nocturnal oximetry; ODIs: mean number of oxygen desaturations ≥ 3% and 4% (ODI3 and ODI4) per hour of analyzed recording; T90: time spent with SpO_2_ < 90%.

**Table 2:** Number and percentage of the subjects reporting each of the symptoms stratified by severity.

Symptom	No SAHS (*n* = 30)	Mild-to-moderate SAHS (*n* = 34)	Severe SAHS (*n* = 40)	*P* value
Snoring (number, %)	27 (90%)	33 (97%)	38 (95 %)	0.468
Gasping (number, %)	4 (13, 3%)	4 (11, 8%)	12 (30%)	0.130
Witnessed apneas (number, %)	1 (3, 3%)	21 (61, 8%)	35 (87, 5%)	0 < 001≠
Nocturia (number, %)	15 (50%)	16 (47, 1)	26 (65%)	0.252
Unrefreshing sleep (number, %)	19 (63, 3%)	20 (58, 8%)	22 (55%)	0.784
Morning headaches (number, %)	6 (20%)	12 (35, 3%)	7 (17, 5%)	0.171
Excessive daytime sleepiness ‡ (number, %)	1 (3, 3%)	1 (2, 9%)	3 (7, 5%)	0.266

^*∗*^ *P* value of the Kruskal–Wallis test comparing subgroup classified for severity. ≠*P* value of no SAHS vs. severe SAHS groups. ‡ = Epworth sleepiness scale ≥15.

**Table 3:** Receiver operating characteristic calculations using the polygraphy apnea-hypopnea index as the reference standard.

	ROC for AHI ≥ 10 AUC 95% confidence limits	ROC for AHI ≥ 30 AUC 95% confidence limits
AHI vs. ODI3	0.941 0.899–0.982	0.911 0.841–0.981
AHI vs. ODI4	0.984 0.964–1	0.922 0.859–0.986
AHI vs. T90	0.759 0.662–0.856	0.653 0.527–0.779

The best discrimination for both mild-to-moderate SAHS and severe SAHS was the ODI3 and ODI4. AHI: apnea-hypopnea index from the polygraphy; AUC: area under the curve; ODIs: mean number of oxygen desaturations ≥3% and 4% (ODI3 and ODI4) per hour of recording; T90: time spent with SpO_2_ < 90%; ROC: receiver-operator characteristic.

## Data Availability

All data that were used to support the findings of this study are included within the article.
